# Biomimetic surface patterning for long-term transmembrane access

**DOI:** 10.1038/srep32485

**Published:** 2016-08-31

**Authors:** Jules J. VanDersarl, Philippe Renaud

**Affiliations:** 1 Microsystems Laboratory, EPFL-STI-IMT-LMIS4, École Polytechnique Fédérale de Lausanne (EPFL), Lausanne, Switzerland

## Abstract

Here we present a planar patch clamp chip based on biomimetic cell membrane fusion. This architecture uses nanometer length-scale surface patterning to replicate the structure and function of membrane proteins, creating a gigaohm seal between the cell and a planar electrode array. The seal is generated passively during cell spreading, without the application of a vacuum to the cell surface. This interface can enable cell-attached and whole-cell recordings that are stable to 72 hours, and generates no visible damage to the cell. The electrodes can be very small (<5 μm) and closely packed, offering a high density platform for cellular measurement.

Patch-clamping is a powerful electrophysiology technique for the study of cell activity, notably ion channel activity and cell polarization, with wide ranging applications throughout drug discovery and basic biology research. Classically, patch clamping is carried out using a glass micropipette with a micron sized opening that is pressed against the cell membrane. Micropipette patch clamping is efficient, but cumbersome, and the highly resistive seal that is required for recording ion channel current is not easy to achieve. In patch recording, maximizing the seal resistance is of critical importance for generating a high quality signal, with a gigaohm (>1 GΩ) resistance being the benchmark for a high quality patch. Whole-cell recording, which is achieved by tearing an opening in the cell membrane via pipette suction, exposes the cell cytoplasm to the liquid-filled pipette electrode, which rapidly disrupts cell integrity and prevents long-term recordings.

In an attempt to improve upon the slow and tedious nature of pipette patch clamping, many researchers have looked towards patch clamp chips, which consist of a multi-electrode array on a cell culture surface (Fig. 1A). Here, the pipette is often replaced by a hole on a chip that is plumbed to vacuum, allowing for easier cell manipulation, automation, and higher throughput. However, the problems of seal quality and loss of cell integrity over time remain. In an even simpler patch chip implementation cells are simply cultured on flat, 2-dimensional electrode arrays. However, with these platforms seal resistances are typically far too low (tens of MΩ^1^) to capture high quality cell data, as the liquid between the cell membrane and the culture surface acts as a current carrier (Fig. 1B).

Although easier and faster to use, patch chips still cannot approximate the idealized patch electrode, which would be an electrode in sealing contact to the cell membrane in cell-attach mode, or an isolated electrode inside the cell for whole cell recording (Fig. 1A). The inability to achieve these geometries with current technology is rooted in the absence of a suction-free, gigaohm (leak-proof) seal.

Recently, researchers have developed clever solutions in an effort to address this need. Many teams have increased cell leak resistances (Fig. 1B, *R*_*seal*_) by making 3-dimensional structures that are engulfed by the cell, including wires^2^,^3^, nano-mushrooms^4^, and pits^5^, which can increase the seal resistance into the hundreds of MΩ^1^. These 3-D structures increase the seal resistance by creating a tortuous path for current traveling in the liquid gap between the basal membrane and the culture surface. However, geometry techniques alone have failed to produce gigaohm seals. These results suggest that a new concept for sealing the gap between the cell membrane and the electrode surround is needed.

Other recent research has demonstrated that it is possible to insert structures into the cell without disrupting the cell membrane. In one implementation, a group successfully created nanopillars that mimic the structure and function of transmembrane proteins^6^. This technique relied on replicating the hydrophilic/hydrophobic/hydrophilic structure of a lipid bilayer by forming a self-assembled monolayer (SAM) of alkanethiols on a thin metal band of gold. This effectively created an artificial transmembrane structure, which could then insert into the cell membrane, forming a stable conduit into a cell.

Unfortunately, the diameter of this artificial transmembrane link is limited to a few hundred nanometers due to the nature of the penetration mechanism^7^. This size is too small to deliver many biomolecules or safely inject large amount of electrical charge into the cells^8^. However, in addition to transmembrane proteins, peripheral membrane proteins can also stably anchor into the outer membrane leaflet via specific hydrophobic/hydrophilic patterning, typically the hydrophobic face of an amphipathic α-helix^9^. This means that a nanopillar (transmembrane structure mimic) is not required for cellular integration; it should be possible to create hydrophobic/hydrophilic line patterns on a flat cell culture surface that mimic a thin α-helix edge, and can integrate into the outer membrane leaflet.

Here we demonstrate that these functionalized planar nanopatterns do indeed integrate into the membranes of cultured cells. To create these patterns we developed a technique that can generate sub 5 nm wide gold (or other material) patterns of various shapes across a 2-D surface via thin film deposition on angled 3-dimensional structures. These thin gold lines are then functionalized with alkanethiols, which insert into the cell membrane when cells are cultured on top (Fig. 1C). When surrounding a microelectrode, this interface is shown to create a tight electric and fluidic seal between the cell membrane and the culture surface. This is demonstrated by maintaining cells in whole-cell mode for three days, something impossible with conventional patch clamping techniques.

## Results and Discussion

### Multielectrode chip

Our planar patch clamp chip is comprised of a multielectrode array (Fig. 2A), which serves as the cell culture surface in the bottom of a well (Fig. 2B). Each electrode is individually connected via a wire trace to a contact pad external the cell culture well. The entire culture surface is passivated by a thin layer of SU-8 to further reduce electric noise and leaks, except for an open section around each electrode (Fig. 2C,D, 20 μm black circles). The electrodes (Figs 2C,D, bright silver discs) are 5 μm in diameter, and are encircled by chrome-gold-chrome metal stacks with a 5 nm gold layer (Fig. 2E). The wire traces are electrically separated from the metal stack by a SiO_2_ layer.

Surface patterning of 5 nm features is extremely challenging with traditional photolithographic techniques, including advanced e-beam lithography. However, thin film deposition can readily be controlled with nanometer resolution in the z-direction, but by depositing the metal stack on angled 3-dimensional structures, we can also use thin film deposition to control dimensionality in the x-y plane (Fig. 3). Other researchers have used thin film deposition to precisely control the dimensions and spacing of nanowires^10^, but this technique requires painstaking physical manipulation of the structures after deposition to make them usable, and can only create 1-D nanowires, not self-enclosed shapes like circles or squares. In contrast, our process is simple, can create patterns with a variety of shapes and sizes, and can be done massively in parallel.

Creating a patch clamp device begins with an electrically insulating substrate (Fig. 3A, brown) topped with the wire trace (Fig. 3B, orange), which defines the electrode pattern and contact pads (titanium and platinum). The entire surface is then sputter coated with amorphous silicon (Fig. 3C, grey), which is patterned with photoresist to define the electrode footprints (Fig. 3D, purple). The resist sidewalls are then angled using thermal reflow (Fig. 3E), which increases thin film coating uniformity on the sidewalls. Using a plasma etcher with chlorine chemistry, the resist and silicon substrate are directionally etched at the same rate, which transfers the photoresist pattern to the α-silicon (Fig. 3F, grey). The substrate is then sputter coated with a passivating SiO_2_ layer (Fig. 3G, blue), a chrome-gold-chrome metal stack (Fig. 3H, yellow), and another SiO_2_ layer (Fig. 3I, blue). The wafer is then processed with CMP (chemical-mechanical polishing), which creates a smooth culture surface, and exposes both the edge of the metal layer stack and the α-Silicon to the cell culture surface (Fig. 3J). Finally, the remaining α-Si is removed with XeF_2_ to expose the platinum electrodes (Fig. 3K). The final structure consists of a recessed metal electrode surrounded by a metal ring (Figs 3L and 2E). The wafer is then diced (Fig. 2A) and a glass ring is fixed to each array to create the cell culture well (Fig. 2B).

### Interface Characterization

Surface membrane proteins most often use the long, thin face of an α-helix to integrate into the cell membrane, and in fact, the long and thin geometry may be a requirement for continuous membrane integration. Artificial techniques for linking lipid bilayers to a solid surface use either proteins^11^ or lipids^12^ that are spaced apart^13^ in a way that they function independently. These anchors can thus secure the membrane to a surface, but cannot create a continuous seal. However, Monte Carlo simulations demonstrate that contiguous alkane anchors can stably insert into a bilayer in the normal direction, but only if they are part of a very thin structure; less than the bilayer thickness^14^. That means that patterning an alkanethiol SAM that integrates into a cell membrane necessitates surface lithography with a resolution below ~5 nm.

The key element in the patch clamp chip is the alkanethiol ring that integrates into the cell membrane, which in turn relies on the metal stack geometry. In order to study this device element, we fabricated simplified test surfaces without electrodes. These test chips were built on standard silicon wafers with a process flow similar to that from Fig. 3, but simplified to remove all steps related to electrode deposition, definition, passivation, and exposure (Fig. S1). The final structure consists of a range of rectangles, squares, and circles, which were successfully outlined with thin metal stacks of various thicknesses (Fig. 4A–D)

Using a fluorescently tagged disulfide molecule (HOOC-C10-S-S-C10-COHN-fluorosceine, ProChimia, Poland)^15^, we selectively labeled the exposed metal lines of the device surface. The gold lines are completely labeled, while the surrounding SiO_2_ and Si remain label free (Fig. 4C,D). This demonstrates both the ability of the above process to make nanometer thick surface features, and also the ability of thiol groups to selectively and completely cover nanometer thick gold lines. Indeed, a TEM EDX (Energy-dispersive X-ray spectroscopy) of a sample cross section shows that the angled sidewalls are completely and uniformly coated with a chrome-gold-chrome layer all the way up to the device top surface (Fig. 4E). Although this relation varies strongly with sidewall angle, coating technique, and coating parameters, here the exposed metal lines (x-y plane) have roughly half the thickness of the deposited film (z-direction). In Fig. 4, the thickness of the gold layer on the angled sidewall is 6 nm after a 10 nm deposition (Figs 4E,F). Due to native oxide formation, the cell culture surface both inside and outside the metal features is SiO_2_ (Fig. S2).

The integration of the cell membranes with the alkanethiol bands was also studied using the test substrates with various sized circles and squares with gold perimeters of different thicknesses. Two different types of cells (HeLa and HEK293) were tested using a similar test strategy: The cell membranes were labeled with a fluorescent (green) tag before being plated on the test substrates. After plating, the cells membranes were labeled with a different type of membrane dye (red), then quickly washed and fixed. While the green membrane dye should stain the cell membrane uniformly (Fig. 5A), the red membrane dye should be delayed in diffusing into the sections of the membrane isolated by the alkanethiol bands (Fig. 5B).

Both the HeLa and HEK293 cells were transiently transfected to express N-terminal GFP-tagged surface membrane protein CMG2^16^. The cells were then plated on the prepared test substrates for 24 hours to allow for adhesion and membrane integration. HeLa cells were washed twice with PBS, stained with 1 μM DiI stain in medium for 5 minutes, then washed twice with PBS and fixed. For the HEK cells, a membrane-anchored extracellular SNAP-tag was induced by adding 5 μg/ml tetracycline to the medium for 24 hours. The cells were then incubated with 5 μM fluorescent SNAP label (SNAP-Surface 549) for 5 minutes before being washed and fixed. The HEK/SNAP staining technique has previously been used to establish membrane continuity around nanofeatures^17^. All cells were imaged in PBS with confocal microscopy to isolate the basal membrane. The confocal images demonstrate that the membrane label (green) added prior to plating is expressed uniformly over the membrane (Fig. 5C), while the membrane dye (red) added after cell attachment is attenuated within the membrane patches isolated by the alkanethiol fences, but is prevalent elsewhere on the membrane (Fig. 5D). The HeLa cells were stained with a lipophilic membrane dye (Dil) that inserts into the cell membrane interior, while the HEK cells labeled a transmembrane receptor protein. Both cell types and labeling methods generated similar results, which illustrates that the alkanethiol-membrane interfacing mechanism is not specific to any single cell line, and that the barrier to dye diffusion is not due simply to impingement of the extramembrane domains of the SNAP protein, but rather some mechanism that extends into the membrane interior.

Prior research on the interaction between alkanethiols and lipid bilayers found that the alkane chain structure has a strong influence on the strength of the alkane-bilayer interaction^18^. The strongest interaction energy is between membranes and short alkane chains (butane and hexane) or longer unsaturated chains, indicating that both hydrophobic and entropic forces contribute to the strength of the interaction. Long saturated chains, which form a crystalline phase, only weakly insert into the bilayer. We tested a variety of chains types at their ability to block membrane dye diffusion, including butanethiol, hexanethiol, octanethiol, decanethiol, dodecanethiol, hexadecanethiol, and tert-dodcanethiol. In agreement with previous work, we found no evidence that saturated alkane chains longer than hexanethiol successfully integrated into the cell bilayer. However, despite lower interaction energy than butanethiol and hexanethiol, we found that tert-dodecanethiol inserted into cell membranes at commensurate rates. This is likely due to the longer tert-dodecane chains having better contact with the cell membrane than the shorter chains, which increase the odds of insertion. Others have found similar results for surface bound RGD anchors, where a longer anchor chain can increase the chances of cell-integrin binding^19^. We also tested gold band thicknesses of 3, 5, and 6 nm. The 5 nm and 6 nm lines performed similarly, while the 3 nm lines failed completely, despite theoretically being the best thickness^14^. This was likely due to the 3 nm lines being too thin to form contiguous lines and/or SAMs with our current process. We found corral size and shape to be irrelevant to membrane integration. FRAP (fluorescence recovery after photobleaching) measurements of diffusion across the alkane chain barrier did not yield definitive results, likely due to the barrier being so thin (see supplemental information).

Images of thiolized rings acting as dye diffusion barriers (Fig. 5D) show that successful events occur in clusters. Vast regions of a chip can show no successful dye barriers, while localized efficiencies, even across many cells, can be close to 100%. This implies that the success or failure of membrane integration can be largely affected by small variations in fabrication or functionalization that still need to be elucidated. These barriers will also likely be much more effective against large membrane constructs, such as lipid rafts, than the small dye molecules tested here.

### Electrical Measurements

Before use, the gold rings on the devices were functionalized with alkane thiols following established protocols (see Methods section). HeLa cells were then plated for 24 hours before electrical testing began. The HeLa cells were DHHC6 depleted, which caused them to spread over a larger surface area than conventional HeLa cells (unpublished observations), which increased the chances of single cells covering an entire electrode. The electrodes are recessed from the cell culture surface by 1–2 μm, but cells grow conformally to such architectures^5^, with the membrane in close contact to the electrode and functionalized band. The cells were then electrically characterized using an Axon Axopatch 200B patchclamp and Axon CNS Digidata 1440A acquisition system with pCLAMP 10 software. The electrical measurements were made in a Faraday cage using a Pt/Ir counter electrode (World Precision Instruments, USA).

The cells interface with the patch clamp chip in a variety of modes (Fig. 6). Using a 5 Hz 20 mV square wave signal, the resistance of an open electrode (Fig. 6A) was determined to be 17.7 ± 2.5 MΩ (Ν = 20). When a cell covers the electrode surrounded with a *non-functionalized* ring (Fig. 6B), the seal resistance ranges from 50–300 MΩ (Ν = 6), a finding consistent with the recessed electrode seal resistances found by others^20^,^21^. When the cell covers an electrode with a *functionalized* gold ring (Fig. 6C), the alkanethiol chains insert into the outer leaflet of the cell membrane, forming a tight electrical seal. In this configuration, the electrical system operates as a high quality cell-attached patch setup with a seal resistance of 5.6 ± 3.3 GΩ (Ν = 14) for successfully sealed cells. Successful seals (>1 GΩ) were achieved over 80% of the time for electrodes completely spanned by a single cell. This seal quality is comparable to conventional glass pipette patch clamps and the best chip based patch clamp systems, while the ability to make such high quality seals over a large orifice without suction is entirely unique^22^.

Traditional patch clamp pipettes transition from cell-attached patching to whole cell clamping through a combination of vacuum and electroporation. This platform does not have the ability to apply a vacuum, but can transition from patch to whole-cell clamping through electroporation alone. With the application of a significant electric field across a cell membrane, the membrane will undergo dielectric breakdown, or electroporation, where pores open in the membrane. Small pores will spontaneously heal, but larger openings, generated by longer and more intense pulse sequences, remain open^23^. Due to the intimacy of the electrode with the cell membrane, the electric field is localized at the cell membrane patch, and a low voltage can induce membrane electroporation. A biphasic voltage pulse sequence of as little as 1.3 V at 200 Hz for 1 second can produce transient pores in the patch, transitioning the cell to whole cell clamping. This transition is characterized by a rise in membrane capacitance (43 ± 22 pF, N = 9), due to the increase in communicating cell membrane surface area, and a decrease in measured resistance (377 ± 179 MΩ, Ν = 9), due to the decrease in *R*_*j*_^24^. The membrane then self-heals and reverts to patch mode within minutes. A longer and more intense pulse train, 3 V at 50 Hz for 5 seconds, generates critically sized pores that do not heal. Here, the cell is in long-term whole cell clamping mode (Fig. 6D). Traditional whole cell clamping deteriorates within minutes or hours, due to homeostasis issues between the cell cytosol and the pipette liquid volume. However, since our configuration has no suction required to keep a seal, no large communicating volume, and a high quality *R*_*seal*_ that prevents fluidic leaks, we can keep the cells in whole cell mode for over 72 hours before complete signal degradation or cell death.

The first whole-cell measurements were taken 15 minutes after electroporation (Fig. 6D), enough time to allow non-critical sized cell membrane pores to heal^25^. Measurements taken at 6 hours (Fig. 6E) and 24 hours (Fig. 6F) also show excellent whole-cell patch characteristics. The capacitance spike is lowered compared to the measurements taken soonest after electroporation, but curve fitting suggests this is due to an increase in electrode/cell membrane resistance, not from a decrease in cell capacitance. This is likely due to longer term membrane healing or electrode fouling. Approximately 1/3 of successfully patched whole cells still show a successful patch 72 hours after electroporation (Fig. 6G), albeit with a degraded signal. Here, the earlier trend of increased membrane/electrode resistance continues, but now with lowered seal (leak) resistance. The decreased seal resistance is likely a sign of a breakdown of the SAM-cell interface. Supporting this breakdown theory is the fact that signals generated from these cells no longer show quality whole-cell or cell-attached patch characteristics (Fig. 6H), despite live-dead staining showing their continued viability. Here the signal (Fig. 6H) more closely resembles that of a cell-electrode pair without the SAM modifications (Fig. 6B) compared to one that does (Fig. 6C). Others have demonstrated that the effect of alkanethiol SAMs on cells in culture is reduced after several days, likely due to some combination of extracellular membrane secretion, cell multi-layering, or SAM degradation^26^. The longevity of these seals could likely be increased with slower growing cell cultures or with a different SAM chemistry.

**Figure 1:**
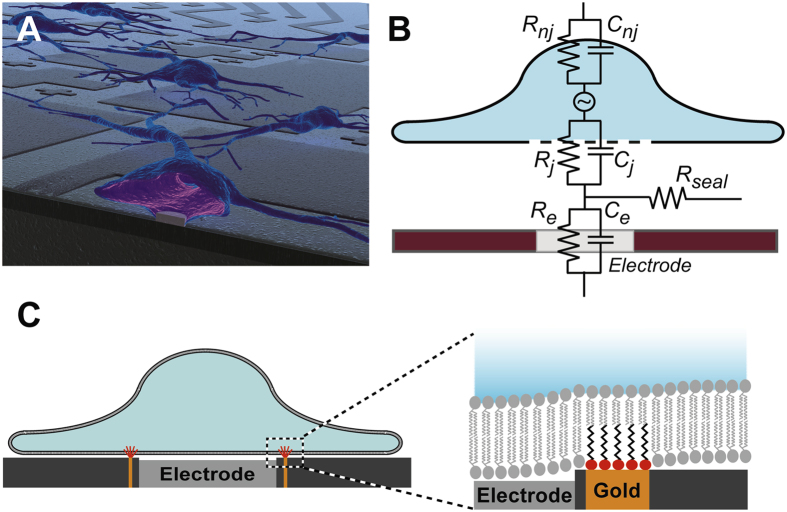
A cartoon of the patch clamp chip concept (**A**). Ideally, for whole cell clamping an electrode would be sealed inside a cell (cell cross section front-center). The electrode/cell interface is defined by several surfaces, each with a characteristic resistance and capacitance (**B**). The leak resistance, *R*_*seal*_, should be as large as possible to increase signal quality and maintain cell homeostasis during whole cell access (other values include the junctional membrane resistance (*R*_*j*_) and capacitance (*C*_*j*_), the non-junctional membrane resistance (*R*_*nj*_) and capacitance (*C*_*nj*_), and the electrode resistance (*R*_*e*_) and capacitance (*C*_*e*_)). A metal band around the electrode, when functionalized with the appropriate alkanethiols, can integrate into the cell membrane (c, cell attached mode). This creates a leak free seal between the electrode and the cell.

**Figure 2:**
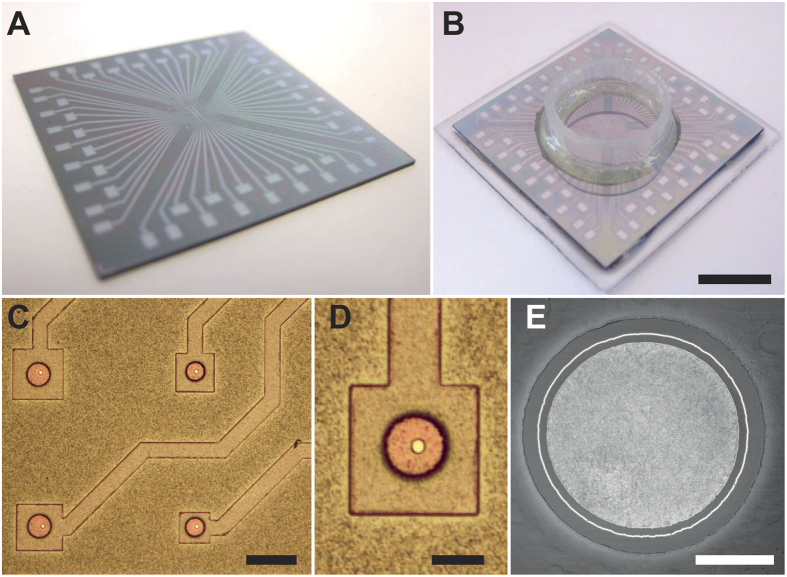
A 60 electrode array is patterned on a silicon chip 30 mm on a side (**A**), which has a glass cylinder epoxied to the top surface to create a cell culture well (**B**). Each wire path connects to only one electrode (**C**). Each electrode pad has an opening in the passivating SU-8 layer (**D**, black circle) around the 5 μm platinum electrode opening (**D**,**E**, silver disc). Each electrode opening is encircled by a thin metal ring only a few nm thick (**E**). The metal ring is separated from the electrode opening by a passivating SiO_2_ layer. Scale bars are (**B**) 10 mm, (**C**) 60 μm, (**D**) 20 μm, and (**E**) 2 μm.

Higher cell-ring integration efficiency was observed for the electrical experiments than for dye experiments, which could be due to the recessed electrode (Fig. 1C) bringing the ring and cell membrane into more intimate contact, or the dye diffusion tests being a more challenging demonstration of integration than the electrical measurements. It is clear that the dye measurements are difficult, as the FRAP experiments and calculations show very large diffusion barriers are needed for a significant change in dye diffusion rate (supplemental information), while it is possible that good electrical seals are formed with non-ideal thiol-membrane seals. A thorough deconvolution of thiol-membrane integration as a function of platform geometry and design parameters (thiol type, surface geometry, band thickness) will need to be refined in future work. Overall, these structures can successfully integrate into cells and form low leak electrical bonds with large electrodes.

## Conclusion

We have demonstrated a new platform for interfacing cells with artificial structures. This system uses alkanethiol SAMs on a 5 nm gold band to mimic the hydrophobic structure of cell membrane integrating proteins. This system can be used to make membrane integrating structures of virtually any shape or size, and thus can be used to interface a cell with large electrodes. As a demonstration of this system, we created a patch clamping chip with gigaohm seals in both cell-attached and whole cell modes. As this platform creates a leak-free seal by inserting into the cell membrane, whole cell patches remain intact for up to 72 hours, a vast improvement over previous work. This method for integrating into a cell membrane is generally applicable, and also has implications for more fundamental research on cell membrane signaling and function. Even stand-alone membrane integrating rings (without electrodes) could be a useful research tool, as they could potentially be used to stabilize *ex vivo* sections of cell membranes for high-throughput drug screening, serving as a more real-life version of the supported lipid bilayer arrays currently in use^27^. Although demonstrated here on only two cell types, a wide variety of cells can potentially be electrically patched with this platform. The electrodes can be very small (<5 μm) and densely packed, which allows for several electrodes under each cell and the monitoring of several cells on the same chip, providing a system that could potentially be used in the study of cellular network interactions. Additional future applications of this tool could include the long-term monitoring of drug induced cell modifications and the continuous voltage clamp of neurons in culture.

## Materials and Methods

### Microfabrication

**Figure 3:**
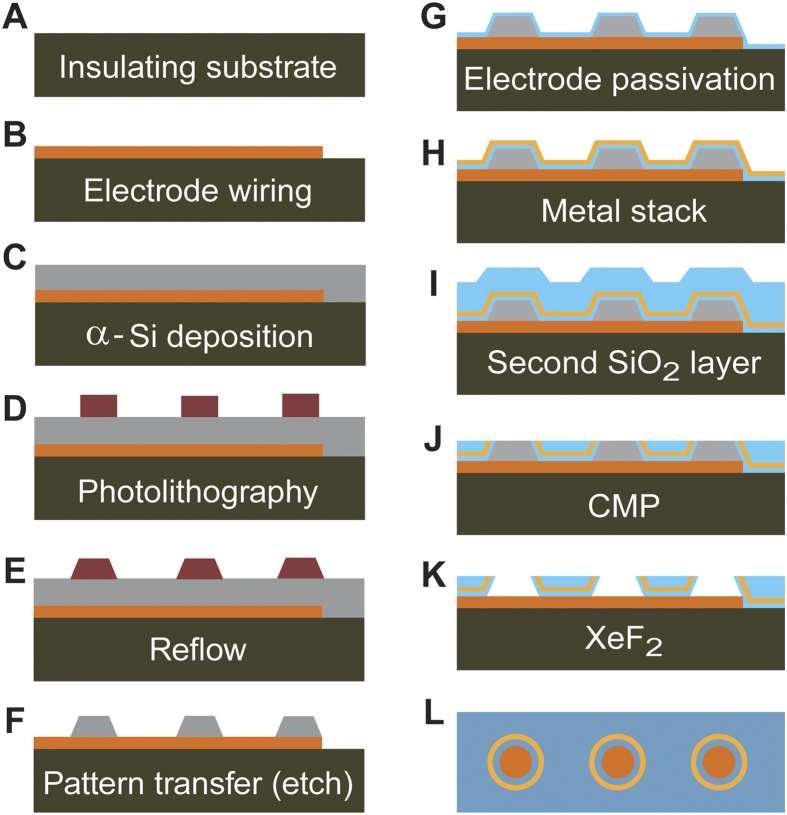
The process flow for creating patch clamp chips starts with an insulating substrate (**A**, silicon wafer with thermal oxide or quartz wafer, brown) which is topped with a photolithographically defined wire trace (**B**, platinum on titanium, orange). The entire wafer is then sputter coated with α-Si (**C**, grey). Positive resist (purple) defines the size and positions of the final patch electrodes (**D**). The resist is then reflowed to create angled sidewalls (**E**), and the wafer is directionally plasma etched to transfer the resist pattern into the underlying α-Si (**F**). A layer of SiO_2_ (blue) passivates the electrodes (**G**), and the sputter deposition of the chrome/gold/chrome metal stack (yellow) creates the surface for eventual alkanethiol functionalization (**H**). The entire device is then backfilled with sputtered SiO_2_ (**I**). CMP removes the excess SiO_2_, exposing the α-Si features and the surrounding metal rings (**J**). A thin layer of SU-8 further passivates the device top surface except for around the α-Si features, reducing the effects of pinhole defects in the SiO_2_ top layer. The α-Si is then selectively etched with XeF_2_ (**K**), and diced to size. A top down view of the final surface shows electrodes (orange) encircled by a thin metal ring (**L**, yellow).

A typical patch clamp chip substrate is a 100 mm silicon wafer with 2 μm thermal oxide (Fig. 3A). 10 nm of Ti and 100 nm of Pt were deposited on the wafer with a Pfeiffer Spider 600 (Pfeiffer Vacuum, Germany) sputter coater, and the wire traces defined by 5 μm of AZ ECI 3027 (MicroChemicals GmbH, Germany) photoresist which served as an etch mask during Cl_2_/Ar plasma etching (STS Multiplex ICP, Surface Technology Systems, UK Fig. 3B). 2 μm of α-Si was then deposited via sputtering on the device surface (Pfeiffer, Fig. 3C). 2 μm AZ ECI resist defines the electrode openings (Fig. 3D), which is then reflowed on a hotplate at 120 °C for 60 s (Fig. 3E). The resist pattern is transferred to the α-Si with Cl_2_ plasma etching (STS Multiplex ICP) until the non-masked α-Si is gone, ~7 minutes. The structures are then sputter coated with 400 nm of SiO_2_ (Pfeiffer, Fig. 3G), sputter coated with the metal stack in a DP 650 (Alliance-Concept, France, Fig. 3H), and backfilled with sputtered SiO_2_ (Pfeiffer, Fig. 3I). The top surface of the α-Si structures are exposed via CMP using a Steag Mecapol E 460 (Steag MicroTech, Germany) at 3psi head pressure with 150H25 Klebosol slurry (Dow Corning, USA) and 85 rpm platen and headspeed for 2–3 minutes (Fig. 3J). The devices were then thoroughly washed and cleaned, and coated with a 5 μm layer of SU-8 for further passivation. The α-Si structures were removed with a homemade XeF_2_ etcher (Fig. 3K), and the wafer was diced to shape (Fig. 3L).

### SAM formation

Patch clamp chips and surface test chips were cleaned with ethanol, dried under nitrogen, treated with oxygen plasma at 100 W for 30 s, and then submerged in a 5 mM alkanethiol solution in 200 proof ethanol overnight (at least 12 hours) for SAM formation. The fluorescent thiols (HOOC-C10-S-S-C10-COHN-fluorosceine, FL 002-m10–0.05, ProChimia, Poland), due to slower disulfide kinetics, were allowed to incubate for at 36 hours. SAM formation took place in an airtight container backfilled with nitrogen, and shielded from light. After incubation, the chips were removed from the thiol solution, rinsed thoroughly in ethanol, sonicated in fresh enthanol for 30 s to remove non-specifically bound molecules, rinsed again with ethanol, and then dried under a nitrogen stream. Cells were cultured on the substrates within 24 hours of SAM preparation.

### DHHC6 depleted HeLa cells

were transfected to express GFP-CMG2 24 hours prior to further testing. Cells were then lifted and plated in 6 well plates with one test substrate in each well bottom. Cells were cultured for 24–48 hours, washed 2X with PBS, stained with 1 μM DiI stain in warm medium for 5–10 minutes, rinsed 2X with PBS, fixed with 3% PFA for 20 minutes, and placed under fresh PBS for imaging.

### HEK293 cells

were cultured in DMEM medium supplemented with 10% FBS, blasticidin, and hygromycin, and were transfected to express GFP-CMG2 24 hours prior to further testing. The cells were then lifted and plated on the test substrates, and a membrane-anchored SNAP-tag was induced on the extracellular membrane through the addition of 5 μg/ml tetracycline to the culture medium. After 24 hours, the SNAP tag were labeled with 5 μM SNAP-Surface 549 (New England BioLabs, USA) for 5–10 minutes in DMEM culture medium. The cells were then washed 2X with PBS, fixed in 3% PFA for 20 minutes, and placed under fresh PBS for imaging.

### Electrical measurements

**Figure 4:**
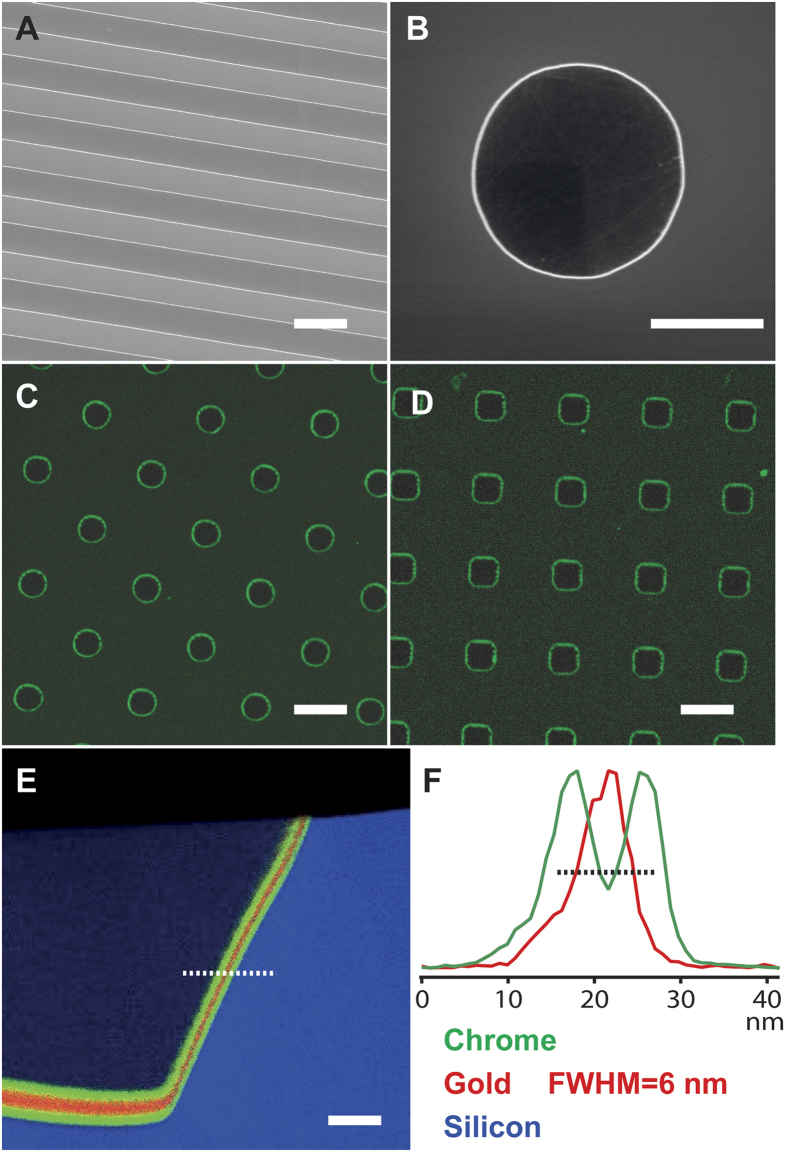
The top surface of the test substrate contains lines (**A**), circles (**B,C**), and squares (**D**) of various size and density. A typical structure consists of a micron sized silicon feature separated from the surrounding SiO_2_ by a thin metal band of chrome/gold/chrome (**B**). The metal band around each feature is in communication with the top surface, and can be selectively functionalized by thiols (**C,D**, green fluorescent thiols). A TEM EDX cross section of the device features shows that gold bands as thin as a few nanometers are continuous and uniform across the entire feature sidewall and up to the top surface (**E**, chrome = green, gold = red, silicon = blue). The cross section view in (**E**) replicates the view depicted in Fig. S1g. Here, a deposited metal thickness of 10 nm corresponds to a sidewall thickness of 6 nm (**F**). The dashed line in (**E**) shows the origin of the EDX data in (**F**). The dashed line in (**F**) depicts the full width at half maximum of the gold peak. Scale bars are (**A**) 4 μm, (**B**) 2 μm, (**C**) 10 μm, (**D**) 10 μm, and (**E**) 40 nm.

**Figure 5:**
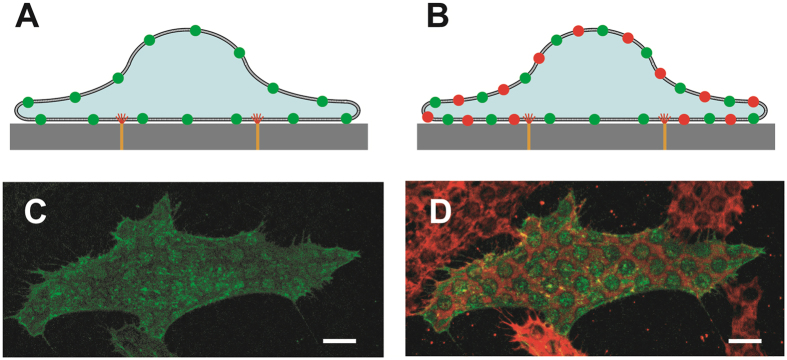
Cartoon of a cell with a GFP membrane label (green) which is then plated on a test structure (**A**). Subsequent addition of a red membrane label is temporarily blocked from diffusing into areas encircled by the alkanethiol fences (**B**). Confocal images of fixed HeLa cells confirm green dye (CMG2 bound GFP) uniformity (**C**) and red dye (DiI) exclusion from the alkanethiol protected disc regions (**D**) in the basal membrane. Cells that were not successfully transfected to express GFP show the DiI stain pattern only (**D**). The location of the alkanethiol bands is visible in Fig. 6C as dark circles due to localized fluorescence quenching. These test platforms used 5 μm diameter circular spot patterns, 5 nm thick gold bands, and hexanethiol. The scale bars are 10 μm.

**Figure 6:**
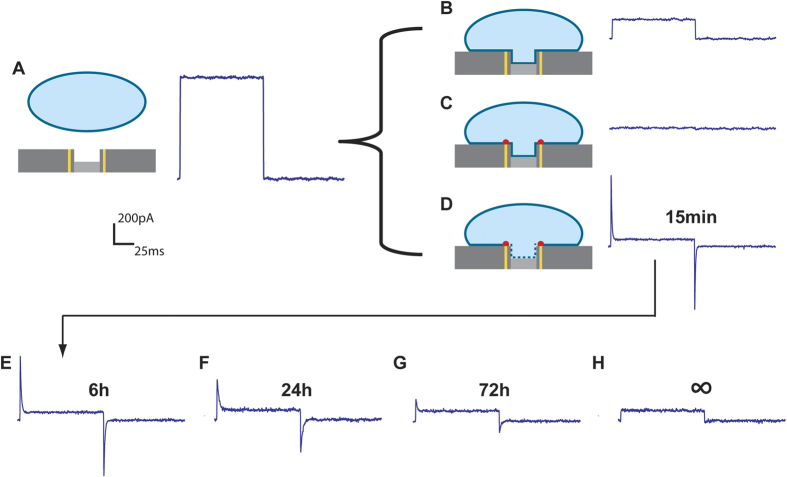
The open electrodes (no cell on top) have a characteristic resistance of ~20 MΩ when tested with a 5 Hz 20 mV (**A**). Cells on top of the electrode can have three different configurations. (**B**) Cells over an electrode surrounded by a non-functionalized ring (gold lines) have a typical seal resistance of ~100 MΩ (**B**), while cells over an electrode with an alkanethiol functionalized ring (gold lines with red spots) have a seal resistance of ~5 GΩ (**C**). Application of an electric pulse sequence permeabilizes the electrode facing cell membrane (dashed line) and generates intracellular electrode access (**D**). This transition is characterized by a decrease in seal resistance (~400 MΩ) and an increase in capacitive signal (~40 pF). The whole cell patch remains intact at 6 hours (**E**), 24 hours (**F**), and even out to 72 hours (**G**) for approximately one-third of successfully patched cells. Cells tested after patch failure (**h**) show current traces more similar to that of an unfunctionalized electrode (**B**) than an unpermeabilized cell (**C**).

were performed with an Axon Axopatch 200B patchclamp with pCLAMP 10 software. Membrane seals were determined using the Membrane Test protocol at 5 Hz with a 20 mV probing signal and a 0 mV hold with 10 sweeps/run. Data was sampled at 10,000 Hz and low pass filtered at 1000 Hz. The current traces shown in Fig. 4 are from sweep number 6/10 from representative cells for each depicted configuration. The current traces shown have been smoothed, but all data analysis was done on unsmoothed traces. Resistances were calculated by fitting the last half of each voltage position, and capacitance measurements were determined from fits of the peaks between the 20% and 80% of the peak height. The system capacitance was compensated to the bare Pt electrode only. Measurements were made in a faraday cage at room temperature with a Pt/Ir counter electrode. HeLa cells were allowed expand for 24 hours before initial testing in DMEM with 10% FBS. Prior to testing the medium was replaced with serum free DMEM during measurements to limit further cell growth and splitting. All statistics reported are from at least 100 measurements and at least 6 different cells and electrodes for each configuration. The pCLAMP software was used to treat and fit the data, and IgorPro was used to smooth and prepare the data for presentation.

### Confocal imaging

was done with a Zeiss LSM 710 upright microscope running ZEN 2010 software and using an Axiocam MRm. The GFP was imaged using a 488 nm laser at 8% power using a 1 airy unit pinhole, while the DiI and SNAP-Surface 549 were imaged with a 561 nm laser at 1.1% power using a 1 airy unit pinhole. All confocal imaging was done with a 63X N-Achromat ceramic water immersion objective with a numerical aperture of 0.90. FRAP was done with this same cmicroscope and objective, but in phenol red free culture solution without FBS. FRAP was done on the membrane bound GFP constructs. After imaging 5 scans of a 4 μm circle, the target area was quickly bleached under high power to obtain between 30% and 60% fluorescence depletion. Recovery of fluorescence was measured at 2 Hz for 60 seconds. The recovery kinetics between bleached regions inside and outside of alkanethiolized rings were compared by fitting exponential functions to the normalized intensity data. The reported FRAP statistics is from 35 measurements from 17 cells.

SEM imaging was performed with a Zeiss LEO 1150. TEM imaging and EDX spectroscopy was performed on a Technai Oriris using sample cross sections prepared with the tripod method.

## Additional Information

**How to cite this article**: VanDersarl, J. J. and Renaud, P. Biomimetic surface patterning for long-term transmembrane access. *Sci. Rep.* **6**, 32485; doi: 10.1038/srep32485 (2016).

## Supplementary Material

Supplementary Information
