# Supplementary material for: Biomimetic surface patterning for long-term transmembrane access
Source: Sci Rep. 2016 Aug 31;6:32485. doi: 10.1038/srep32485 (PMC5006161; doi:10.1038/srep32485)
Supplement: Supplementary Information [file srep32485-s1.pdf]

Biomimetic surface patterning for long-term transmembrane access

Jules J. VanDersarl[1] and Philippe Renaud[1]*

Author affiliations: [1]Microsystems Laboratory, EPFL-STI-IMT-LMIS4, École Polytechnique Fédérale de Lausanne (EPFL), Lausanne, Switzerland

*Correspondence to: philippe.renaud@epfl.ch

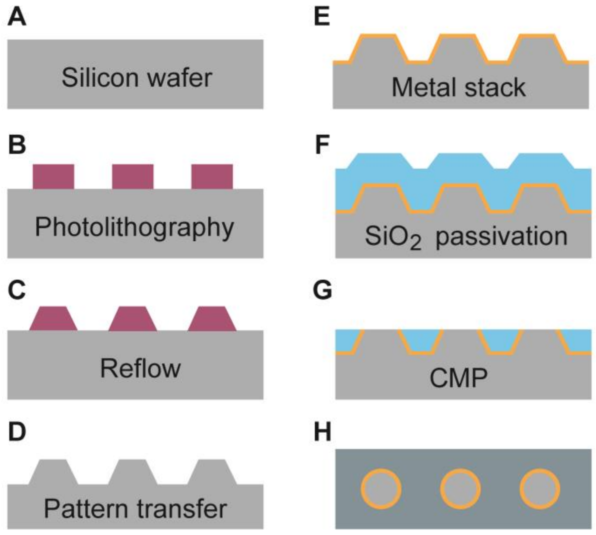

Figure S1:

The test platform process flow is a simplified version of the patch clamp chip process flow (Figure 3). The process starts with a bare silicon wafer (a) which is patterned with photoresist to define the spot pattern (b). The resist is then reflowed (c) before a directional plasma etch transfers the resist pattern into the silicon substrate (d). A thin metal stack of chrome/gold/chrome is uniformly deposited over the entire surface via sputter deposition (e), and then followed with sputter deposition of SiO_2 (f). Finally, CMP removes the top layer of SiO_2 and the metal stack over the 3D structures (g), creating isolated spots ringed by a thin metal line (h, top view of device).

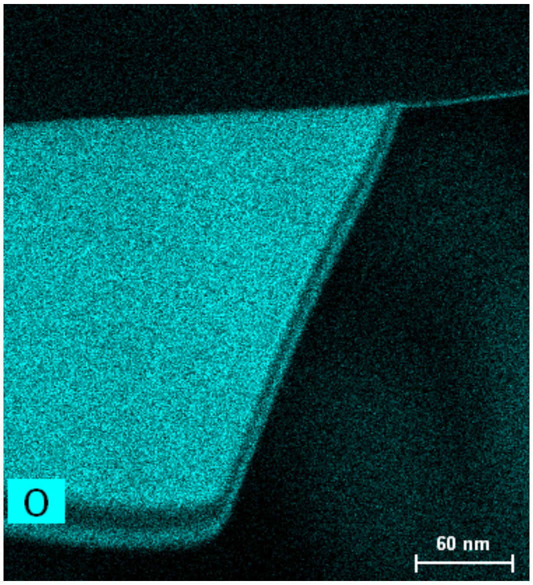

Figure S2:

The oxygen channel of the EDX TEM image shown in Figure 5e. The backfilled SiO_2 is clearly evident outside of the 3D silicon features (left). Oxygen is evident at the silicon cell culture surface (right top), due to native oxide formation. Cells cultured on the test substrates have a similar culture surface on both sides of the metal/akanethiol lines.

Supplemental Information:

Figure 6d shows membrane dye diffusion is blocked from entering the thiol functionalized rings on fixed cells. On non-fixed cells, the dye will eventually diffuse through the thiol barriers. We studied the strength of this barrier to diffusion with FRAP (fluorescence recovery after photobleaching) on the basal leaflet of HeLa cells with GFP labeled CMG2 on the cell surface. A 4 μm bleached spot outside a thiolized ring recovers half its fluorescence (t_{50}) in 11.0±2.6 seconds, which corresponds to a uniform membrane diffusion constant of 0.4 $\mu m^2/s$. A 4 μm bleached spot inside a functionalized ring has a t_{50} of 13.1±4.8 seconds. Although this is only a 19% increase in average t_{50} recovery time between the functionalized and non-functionalized rings, this equates to the thiol band region having a diffusion constant more than 100X lower than that of the bulk membrane. The small change in FRAP recovery time equating to a large change in diffusion rates across the thiol band is due to the band being so thin (3-5 nm) compared to the overall FRAP area.
